# Progress in Cardiac Resynchronisation Therapy and Optimisation

**DOI:** 10.3390/jcdd10100428

**Published:** 2023-10-17

**Authors:** Zaki Akhtar, Mark M. Gallagher, Christos Kontogiannis, Lisa W. M. Leung, Michael Spartalis, Fadi Jouhra, Manav Sohal, Nesan Shanmugam

**Affiliations:** 1 Department of Cardiology, St George’s University Hospital, Blackshaw Road, London SW17 0QT, UK; 2 Department of Cardiology, National and Kapodistrian University of Athens, 10679 Athens, Greece

**Keywords:** device therapy, CRT, pacemaker, heart failure

## Abstract

Cardiac resynchronisation therapy (CRT) has become the cornerstone of heart failure (HF) treatment. Despite the obvious benefit from this therapy, an estimated 30% of CRT patients do not respond (“non-responders”). The cause of “non-response” is multi-factorial and includes suboptimal device settings. To optimise CRT settings, echocardiography has been considered the gold standard but has limitations: it is user dependent and consumes time and resources. CRT proprietary algorithms have been developed to perform device optimisation efficiently and with limited resources. In this review, we discuss CRT optimisation including the various adopted proprietary algorithms and conduction system pacing.

## 1. Introduction

It is estimated that over 920,000 people in the United Kingdom are living with heart failure (HF), an increase of 23% between 2002–2014 [1,2]. An ageing population and improved survival from cardiovascular diseases are contributing to a steady rise in the prevalence of this condition [1]. Established therapy consists of both pharmacological and non-pharmacological options. Optimal medical therapy includes beta-blockers (BBs), angiotensin-converting enzyme inhibitors (ACE-is)/angiotensin receptor blockers (ARBs), mineralocorticoid receptor antagonists (MRAs), a neprilysin inhibitor combination with ARB (sacubutril/valsartan) and sodium glucose co-transporter-2 (SGLT-2) inhibitors, all of which have proven beneficial [3,4,5,6,7]. 

Non-pharmacological strategies target HF patients that meet certain criteria; however, their use has been widely spread [8]. Interventions for ischaemic and valvular heart disease as well as implanted devices that aim to limit the risk of sudden cardiac death (SCD) are often necessary. Additionally, in a specific subgroup of HF patients, cardiac resynchronisation therapy (CRT) has been recognised to improve performance status and mortality risk by correcting the dyssynchrony produced by the conduction system disturbances that often accompany heart failure [9].

Almost 30% of HF patients have abnormal intraventricular electrical conduction, manifesting as a prolonged QRS duration [10] commonly in the form of a left bundle branch block (LBBB). Mechanically, this translates to a delay in contraction between parts of the myocardium [11]. In the presence of an LBBB, left ventricle (LV) contraction occurs later than the right ventricle (RV) [12] and there is a delayed activation of the LV lateral wall compared to the other segments [13]. This dyssynchrony has a negative impact on the cardiac cycle, shortening the diastolic filling time and exacerbating the interventricular septal motion resulting in an inefficient LV contraction and a reduced ejection fraction (EF). Both the systolic and diastolic function are consequently impaired [12]. Abnormalities of atrioventricular (AV) conduction are also common in heart failure; the correction of these by pacing in the traditional manner with a single lead in the right ventricle produces ventricular dyssynchrony, similar to that of LBBB. 

## 2. Cardiac Resynchronisation

### 2.1. Cardiac Resynchronisation Therapy

Early small studies of cardiac resynchronisation indicated a positive effect in selected patients, by improving their haemodynamic and functional status [14]. This was confirmed by large clinical trials, which demonstrated the benefits of CRT on patients with LV systolic impairment and conduction defects. In the MIRACLE study, a randomised controlled trial of patients with HF and a severe conduction delay (QRS > 130 ms), the use of CRT was associated with a significant improvement in the quality of life, exercise capacity and LV ejection fraction (LVEF) [15]. These benefits were validated by the CARE-HF, COMPANION and MADIT-CRT trials that further confirmed a significant reduction in the overall risk of death as well as hospitalisation secondary to HF events [16,17,18]. More recently, the BUDAPEST CRT Upgrade trial has validated CRT in patients with a reduced LVEF and a significant RV pacing burden. In this multi-centre randomised controlled trial, 360 patients with symptomatic HF (LVEF ≤ 35%) and a >20% pacing burden were randomised to either undergo an upgrade to a CRT or an implantable cardioverter defibrillator. The authors found that, in patients with pacing-aggravated LV dysfunction, CRT reduced the risk of all-cause mortality and HF hospitalisation and improved LV remodelling [19]. 

In HF patients with an LVEF of >35%, the need for CRT is less obvious. In a retrospective analysis of PROSPECT, Chung et al. identified a cohort of HF patients with an LVEF of >35% but that otherwise fulfilled the CRT criteria. This group of patients derived benefit from CRT, similar to patients with an LVEF of <35%, hypothesising the possibility of CRT in symptomatic HF patients with an LVEF of >35% and a conduction delay (QRS ≥ 130 ms) [20]. To date, there is no randomised trial to test this hypothesis, probably due to poor recruitment; electrical dyssynchrony may be less prevalent in patients with an LVEF of >35% [21]. 

### 2.2. Responders and Non-Responders

Despite the compelling evidence for the benefits of CRT in the overall population of HF patients with conduction defects, up to 30% of patients with these characteristics do not respond. The term “non-responder” is widely used, but no universal definition exists. The term implies the existence of a binary phenomenon, with most patients receiving distinct and uniform benefits, whilst some experience none. Evidence for such a clear dichotomy is limited, but it is certain that some patients receive substantially more benefits than others; it is important to understand this phenomenon, in order to direct therapy accurately to patients that are most likely to benefit from CRT.

The causes of “non-response” to CRT is likely multifactorial, and historical pre-implant predictors include ischaemic cardiomyopathy, extensive myocardial scarring, a lack of mechanical dyssynchrony, a narrow QRS and an absence of LBBB [11,22]. More recently, the pre-CRT QRS area has also been identified as a predictor of outcome. This index is based on the calculated area under a three-dimensional QRS which represents the vector of electrical forces during ventricular depolarisation, whereby high values indicate electrical dyssynchrony and an activation delay. A multicentre retrospective analysis of 1492 patients found that a QRS area of >109 μVs may be associated with improved remodelling and clinical outcomes. As a predictor of outcome, whilst the QRS area was non-inferior to the combined factor of LBBB and QRS duration, in patients with non-LBBB morphology and a QRS duration of >150 ms, this vectorcardiographic index is more compelling [23]. To improve the predictability of response, Maass et al. evaluated a number of variables in a multicentre prospective observational study consisting of 240 patients. The authors found that CRT patients with a younger age, larger QRS area, longer interventricular delay and apical rocking (CAVIAR score) were most likely to demonstrate LV reverse remodelling [24]. 

Following CRT implant, significant factors have been identified by Mullens et al., which may contribute to non-response. In a study of 75 patients with CRT who did not exhibit a positive response, the most frequently attributed cause was suboptimal device settings (45%) followed by inadequate medical therapy (32%), arrhythmias (32%) and an inappropriate lead position (21%) [25]; these results suggest that “non-response” is pre-dominantly attributable to the defects in the therapy delivered.

### 2.3. Measuring the Response to Resynchronisation

There is no gold-standard method for measuring the response to CRT. Echocardiographic measurements of LV performance are the most widely used endpoint but with inherent limitations. With this method, a change in LV systolic function is determined pre- and post-therapy, and an improvement in the LVEF is interpreted as a positive response. Reverse remodelling is another marker of response and is determined by a reduction in chamber size and volume [26]. There is a significant association between left heart reverse remodelling and outcome in CRT patients. Mathias et al., in a sub study of MADIT-CRT, found that a complete reverse remodelling of the LV and left atrium (LA) was associated with significant long-term HF and mortality benefits and the absence of any reverse remodelling indicated poor outcomes [27]. Kloosterman et al. validated these findings in a retrospective analysis of 365 patients and also concluded that LA reverse remodelling was only associated with an intermediate outcome; clinical improvements were noted. The LA size and volume may be a surrogate marker for LV filling pressures with reverse remodelling, indicative of an improvement in LV filling and diastolic function [28]. These long-term outcomes are objective endpoints, but echocardiography in most cases is two-dimensional, user-dependent, exposed to observer variability and has limited evidence to demonstrate the predictability of response [29].

The LV *dp/dt max* has been used as a measure of acute haemodynamic response in CRT. It represents the rate of LV pressure increase and is considered a surrogate marker of LV contractility [30]. Non-invasive measurements using echocardiography require the presence of a measurable mitral regurgitation (MR) jet, whilst invasive recording is achieved using a high-fidelity pressure wire positioned temporarily in the LV. This parameter demonstrates an immediate haemodynamic response to CRT, but whether this translates into long-term outcomes remains debatable. A canine model evaluating the effects of CRT on haemodynamics demonstrated an immediate improvement in the LV *dp/dt max*; however, the authors concluded that LV end systolic volume was a better indicator of acute haemodynamic response [31]. 

The simplicity of executing and interpreting the 6-minute walk test have made this an important tool in quantifying the response to CRT. Originally designed for use in patients with chronic respiratory disease, the distance walked in 6 minutes has been found to be a predictor of morbidity and mortality in patients with chronic heart failure [32]. As a surrogate marker of functional capacity, patients who covered <300 m were more likely to be hospitalised and die than patients who walked >450 m [32]. There are obvious limitations including a difficulty in reproducibility: patients tend to perform better on repeat attempts if performed within 30 min of the first [33], and their exercise capacity can be affected by external variates, such as motivation and encouragement, and by non-cardiac conditions, such as arthritis.

The measurement of functional capacity with Volume of Oxygen uptake (VO^2^max) has also been utilised to evaluate CRT response [15]. Through the physiological measurement of gaseous exchange, this test presents an objective index of functional capacity and potentially is a better predictor of prognosis in heart failure than the 6-minute walk test [34]. However, this test is expensive, with limited availability, and patients will need to be mobile enough to exercise on a treadmill.

The subjective response to CRT has been difficult to measure accurately as it is dependent on patient feedback. The New York Heart Association functional class remains a popular method as it encapsulates all reported patient symptoms into a single numerical variable. Other questionnaires, such as the Minnesota Living with Heart Failure Questionnaire, evaluate patients’ quality of life as a measure of functional limitations. These methods do not take into account short-term clinical deterioration before the assessment. Packer’s clinical composite score was developed to evaluate patient response to drug or device therapy for heart failure. It is based on patient functional status and patient global assessment, whilst accounting for changes in capacity during the follow-up period [35].

## 3. Optimisation

### 3.1. Echo Optimisation

Optimal AV delay allows for a maximal diastolic filling time (DFT) before the onset of LV contraction. Based on past studies, this is commonly set empirically at 120 ms [36], but echocardiography-based techniques have been developed to attempt patient-tailored optimisations. The Iterative method utilises PW doppler during echocardiography to measure DFT (E wave and A wave). A long AV delay is initially programmed but decremented in 20 ms steps until the A wave truncates, at which point it is increased in 10 ms steps to achieve the shortest AV delay without A-wave truncation [37]. The CARE-HF trial incorporated this methodology for all patients, and although there were obvious benefits in symptoms, quality of life and survival compared to medical treatments, the trial was not designed to compare methods of CRT optimisation [18]. 

A retrospective study by Kedia et al. examined patients undergoing CRT optimisation, compared Ritter’s method (which was applied in the majority of the patients) to the Iterative method. In this study, with no control group, the single attributable benefit of optimisation was an improvement of the diastolic function, observed only in 9% of the patients [38]. Evidence clarifying its benefits remain scarce.

Ritter’s method was validated from dual-chamber pacemaker studies in patients with preserved LVEF [39]. This requires a programming of two extreme delays, short (AV_short_) and long (AV_long_) intervals, and for each, a calculation of the time between QRS onset and the completion of the A-wave truncation. The optimal AV delay is then determined using the following formula: AV_optimal_ = AV_short_ + [(AV_long_ + QA_long_) − (AV_short_ + QA_short_)]. This calculates the longest AV delay without disrupting the A wave. Whether this formula actually corresponds to the optimal AV delay among patients with HF and CRT is open to debate. A small study measuring invasive LV *dp/dt max*, compared Ritter’s method with other optimisation techniques (a device-based algorithm and aortic velocity-time integral (VTI)) and found it to be the least effective [40]. Conversely, Melzer et al. compared Ritter’s method with radionuclide ventriculography in patients with dual-chamber pacemakers and found a good correlation between the two in localising the optimal AV delay, especially in patients with an impaired LV systolic function [41].

Mitral inflow VTI is a marker of the LV filling volume, provided the mitral valve area remains stable. In a study of 30 patients, this was compared with other echocardiographic measurements (Aortic VTI, Ritter’s method and DFT) and correlated with invasive *dp/dt max* [42]. The most accurate “prediction” of optimal AV delay, as estimated by invasive *dp/dt max*, was performed by mitral inflow VTI (97%), whilst Ritter’s method fared the worst. Left ventricle *dp/dt max* is an index of LV contractility; taking into consideration that the heart rate and contractility remain unchanged, this parameter is affected by the preload through the Frank–Starling mechanism. Therefore, it is no surprise that the mitral inflow VTI, a measure of preload, can manipulate the LV *dp/dt max* [42].

### 3.2. Optimising Lead Position

It has been suggested that operators should seek the area of latest activation of the left ventricle as the target site for the LV lead. One measure used to identify the optimal site is the index QLV, defined as the interval from the onset of the QRS on the surface ECG to the centre of the largest deflection on the local unipolar electrogram measured from the left ventricular lead. The QLV interval may represent the electrical delay witnessed in HF; the latest activated segment would have the longest QLV. In a sub study of SMARTAV, Gold et al. found that LV pacing at the longest QLV site was associated with reverse remodelling and an improvement in the quality of life [43]. Speckle tracking echocardiography is an alternative approach to identify the site of the latest mechanical activation, which could be used as a template to optimise the LV lead implant by operators. The STARTER and TARGET randomised controlled trials found that pacing from the echocardiographic-identified optimal site significantly improved mortality and HF-related hospitalisations for CRT patients compared to routine LV lead implants; the optimisation of LV lead positioning was associated with better patient outcomes [44,45]. However, a percutaneous LV lead implant at the optimal site may not be achievable in all cases, potentially stifling the clinical response. Surgical epicardial LV lead placement is feasible and potentially better for siting; this implant is not restricted by venous anatomy. A recent retrospective analysis has also suggested that surgically sited epicardial leads may perform better due to a lower risk of displacement, lead fractures and PNS that plague traditional transvenous leads. This analysis did not include the performance of modern quadripolar LV leads, and therefore its applicability may be limited to the older generation of leads [46]. 

**Figure 1:**
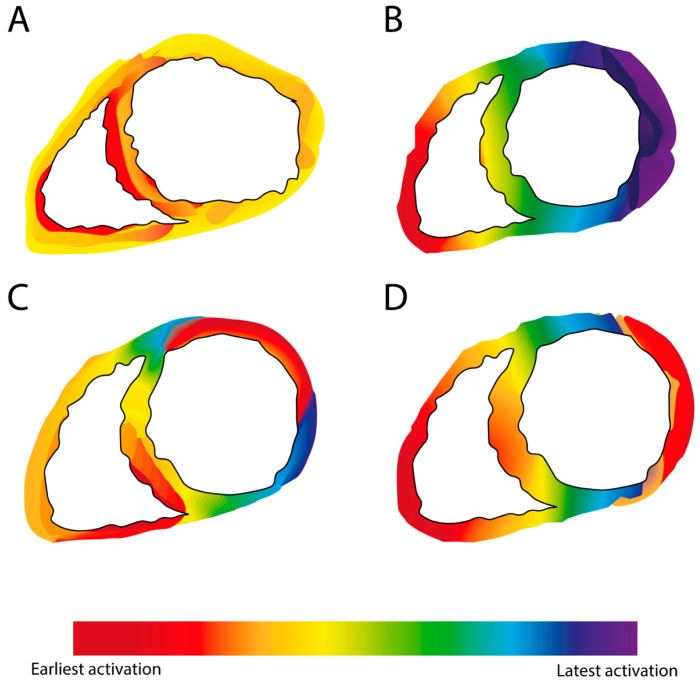
A re-imagined myocardial activation map. (**A**) Normal heart, whereby the earliest activation sequence originates at the septum and propagates quickly across the healthy myocardium. (**B**) In heart failures with a left bundle branch block, the earliest activation is seen at the right ventricle and the depolarisation wavefront propagates across to the left ventricle in a heterogenous fashion; the latest-activated segment is the most lateral portion of the left ventricle. (**C**) Cardiac resynchronisation therapy with the left ventricle activated at the high lateral region and the right ventricle directly opposite at the low septum. Despite the resynchronisation, heterogeneity in the left ventricle activation persists. (**D**) Cardiac resynchronisation therapy with a more traditional positioning of the leads. The left ventricle is activated from the most lateral site, whilst the right ventricle is activated at the apex.

**Figure 2:**
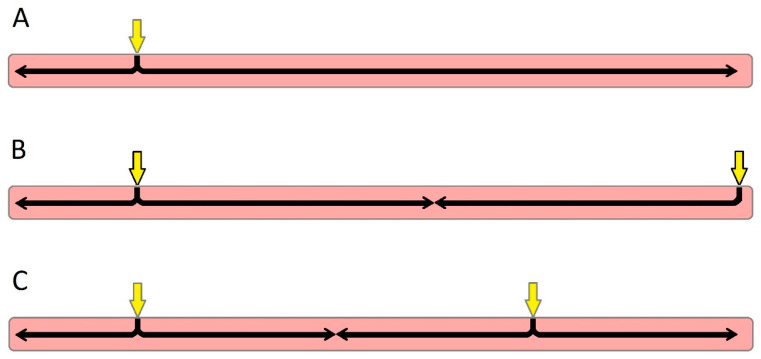
A reductionist model of ventricle activation during pacing. (**A**) Activating the right ventricle at a single point (yellow arrow) results in a depolarisation wavefront propagating quickly throughout the right ventricle but taking longer to activate all of the left ventricle (black arrows). (**B**) Stimulating the left ventricle at the point that is activated latest during right-ventricular pacing reduces the time taken to depolarise all the myocardium. The length of the longest black arrow is halved compared to panel (**A**), implying a halving of the interval. (**C**) An even better degree of synchronisation is achieved by stimulation at a point that is not the latest activated in right-ventricular pacing, but a point located between that latest point and the point of stimulation in the right ventricle. The longest black arrow is now a third of that in panel (**A**).

Using electrocardiographic imaging (ECGI) on 111 CRT patients, Parreira et al. demonstrated that pacing the LV within 47 mm of the latest LV electrical activation site was associated with a reduction in the LV end systolic volume; otherwise, there was no significant response [47]. On theoretical grounds, this principle has potential limitations. Pacing the LV at the latest-activated segment resets this as the earliest site, rendering other segments as the “late activation” [48] (Figure 1), and some dyssynchrony persists as LV conduction remains heterogenous. ECGI during biventricular pacing in 10 patients demonstrated this phenomenon; pacing of the lateral wall resulted in this segment being the earliest activated with heterogenous activation times surrounding this segment [49]. It is plausible that the optimum LV site is that which, in combination with RV pacing and/or intrinsic conduction, makes all areas depolarise, as close as possible to, simultaneously (Figure 2). No single simple rule can identify this site. 

The site of latest activation may also be unsuitable because of the factors that underly this lateness: functional block and localised fibrosis may contribute to this making it a mechanically unproductive site and one from which outgoing depolarisation activation must be set much earlier than the RV to achieve uniform biventricular contraction [50]. 

While the optimum site of LV stimulation is often debated, the optimum choice of the RV stimulation site receives less attention. A common and pragmatic approach to site selection is to position the RV and LV leads as far apart as possible when viewing the LV from the apex, based on the idea that the depolarisation wavefront should propagate smoothly through the myocardium, achieving symmetry (Figure 2); this assumption is untrue, but may be a close enough approximation of the truth to retain some value. It has the merit of operational simplicity, facilitating rapid decision making by the implanting physician without the need for multiple measurements during the procedure or for extensive pre-implant investigations and planning.

### 3.3. Optimising the Atrioventricular Interval

To achieve the maximal benefits of CRT, pacing indices (AV and interventricular (VV) delay) must be individually tailored. Atrial contraction contributes up to 30% of ventricular filling in patients with ventricular dysfunction, highlighting the importance of finetuning the AV timing [51]. Early small studies based on dual-chamber pacemakers used in patients with heart failure demonstrated that an optimal AV interval can have a positive effect on the LV filling time, improving the stroke volume, haemodynamics, exercise capacity and oxygen consumption [52]. This was followed by the landmark CRT trials, which adopted AV delay optimisation [15,17,18].

**Figure 3:**
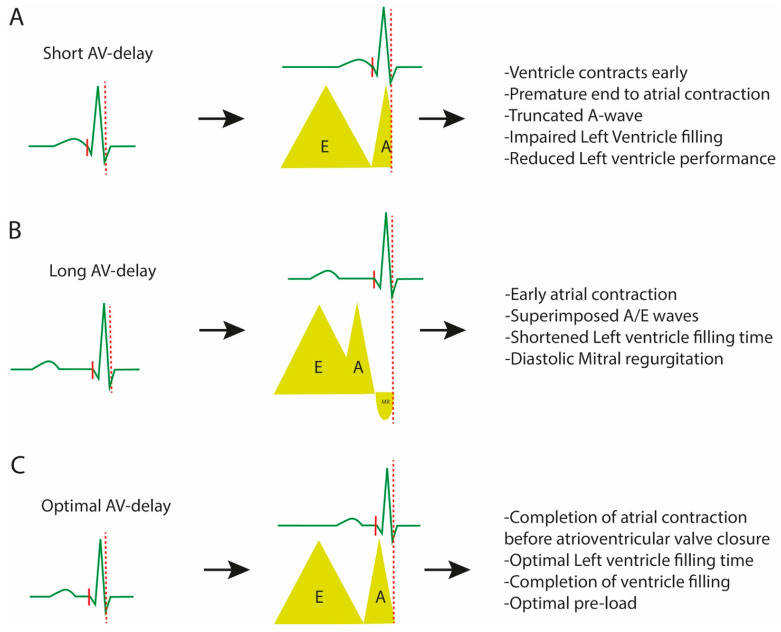
Diagram depicting echocardiogram AV optimisation. (**A**) Short AV delay results in early ventricular contraction with truncation of the A wave on pulsed wave doppler. This results in a premature end to atrial contraction and an impaired LV filling and thus a reduced LV performance. (**B**) Long AV delay results in an early atrial contraction with shortened LV filling time; on the pulsed wave doppler, the A/E waves are super imposed with diastolic mitral regurgitation. (**C**) The optimal AV delay results in a completion of atrial contraction in a timely fashion with a closure of the mitral valve at the end of the A wave and an absence of diastolic mitral regurgitation.

Optimisation of the AV delay should maximise LV filling. When the AV interval is too short, mitral valve closure occurs before the completion of the LA contraction, limiting the atrial contribution to LV filling, seen as a separation of the E and A waves on the mitral inflow pulsed wave (PW) doppler with truncation of the A wave (Figure 3A). On the other hand, an overly long AV interval results in an early LA contraction terminating before LV filling completes, observed as a fusion of the E and A waves on the mitral inflow PW doppler. This results in a premature end to the LA contribution to LV filling and a wasted diastole with a diastolic MR (Figure 3B) [51,53]. Both of these scenarios reduce LV filling and impair the stroke volume and thus cardiac output. The optimal AV delay is therefore the interval that allows for an uncompromised, maximal LA contribution to LV filling, before the onset of the LV contraction (Figure 3C).

### 3.4. Optimising the Inter-Ventricular Interval

In HF patients with LBBB, the RV activates before the LV, and the septal segment of the LV activates before the lateral wall. This may be counteracted by stimulating the LV slightly earlier than RV activation to try and achieve synchronisation between the ventricles and within the LV. Positioning of the LV lead is traditionally aimed at the latest activated cardiac segment to overcome this interventricular delay. The extent of benefits could be influenced by the severity of the pre-existing abnormality of cardiac conduction and contractility, by the positioning of both leads and by the interval between stimulation in each ventricle (VV interval), yet the majority of the large trials investigating the benefits of CRT have incorporated AV optimisation [15,17]. 

There is evidence that optimising the VV delay is beneficial: Van Gelder et al. used invasive LV *dP/dt max* to demonstrate that VV optimisation provided benefits supplementary to optimal AV delay, resulting in an acute improvement of the haemodynamics [54]. Clinically, this was supported by the findings of the RESPONSE-HF trial, which measured the clinical response by the use of the 6 min walk test and the New York Heart Association (NYHA) functional class. In this study of 816 patients having had CRT, 65 were identified as “non-responders”, classified as an absence of improvement in the NYHA class and 6 min walk test after 3 months of therapy. These patients were randomised to either a continuation of simultaneous biventricular pacing or sequential biventricular pacing with VV optimisation. At a 6-month follow-up, the optimised group demonstrated a numerically higher response compared to the simultaneous biventricular pacing cohort (68.9% vs. 50%, respectively) [55,56].

Other small studies have also found improvements in the haemodynamics and LV function with sequential biventricular pacing. Perego et al. demonstrated, in a study of 12 patients, a bigger improvement in LV *dP/dt max* with sequential (optimised VV delay) than with simultaneous biventricular pacing [57], and Sogaard et al. reported a significant improvement in the LV function with sequential biventricular pacing (optimised VV delay) in 20 consecutive patients, which translated to an improvement in the 6 min walk test and NYHA functional class [58]. It is hypothesised that simultaneous biventricular pacing does not consider the activation delay witnessed in the epicardial stimulation of the LV and the endocardial triggering of the RV. This will be compounded further by the heterogeneity in ventricular lead positioning between patients. It is thought that sequential biventricular pacing with the co-ordinated activation of the ventricles accounts for this and truly inspires a synchronous biventricular contraction [57]. Conversely, a large randomised trial (DECREASE-HF) indicated that sequential biventricular pacing with programmed LV and RV activation times was similar to simultaneous biventricular pacing, suggesting that changing the VV delay interval may not have any significant additional benefit for most CRT candidates [59]. The use of this optimisation method is therefore reserved for the “non-responder” group [55]. 

## 4. Device Optimisation

### 4.1. Multipoint Pacing

Heterogeneity in the location of conduction block, as well as in the site and burden of LV scar, presents challenges to optimal resynchronisation and may contribute to the variability of “response” [60]. LV pacing from multiple sites has been suggested as a way to maximise the likelihood of attaining the optimal pacing site. 

Multi-site LV pacing provides excitation of a broader LV area that may improve the likelihood of capturing the region of latest activation and mimic the physiological excitation more closely than single-site LV pacing. The effects of pacing from distinct LV sites may be particularly important in the presence of LBBB to overcome the challenge of the intramyocardial line of blocks seen in this condition [61]. Early trials exploring dual-site LV pacing using separate leads demonstrated significant reverse LV remodelling [62]; adding a third LV lead to expand the capability of multi-site pacing was feasible but technically challenging with high procedural complication rates [63]. An alternative method of achieving stimulation at multiple LV sites has been applied using multipoint pacing (MPP) from a single LV multipolar lead.

Quadripolar leads have been used increasingly since the first reported implantation in 2010, providing the advantage of up to 17 pacing vectors [64]. Numerous studies have reported on the safety and efficacy of these leads, demonstrating good rates of successful implantation and stability, with acceptable displacement rates [65]. These leads offer an immediate benefit over older bipolar or unipolar leads: the greater variety of pacing configurations permit programmable solutions to high pacing thresholds and phrenic nerve stimulation (PNS) [64], where conventional LV leads might require physical re-siting. Using MPP, Ohlow et al. achieved satisfactory LV pacing without PNS in 92% of cases [66], a significantly greater rate of successful implantation compared to bipolar leads in the same vein. 

Multipoint pacing can result in an immediate improvement in the haemodynamic response. Thibault et al. presented an acute improvement in LV performance measured in the form of LV *dP/dt max* with stimulations from the most proximal and distal points [67], whilst Pappone et al. proved a significant effect on acute haemodynamics (stroke volume and stroke work) over a complete cardiac cycle using the pressure–volume loop analysis [68]. Clinically, the IRON-MPP study found an improvement in the LV function over a 6-month period with this modality. In this prospective observational registry, patients with CRT exhibited a favourable effect on the LV function, with an enhanced benefit with MPP compared to conventional CRT (LVEF 39.1% vs. LVEF 34.7%, *p* < 0.01); an early activation of the MPP function was a significant predictor of LV function improvement in this study (OR 2.5, *p* < 0.01) [69].

#### Optimising MPP

Patient selection appears to maximise the gain from MPP. In their study of 16 patients, Sohal et al. found that multi-site pacing may benefit patients with a non-strict LBBB criteria and minimal scar; patients with a strict LBBB coinciding with no scar did not gain any additional benefit compared to conventional CRT [70]. In the MPP IDE trial, patients randomised to MPP received an MPP programmed with a wide LV electrode anatomical separation (≥30 mm) plus a short timing interval (MPP-AS), or settings at the operator’s discretion (MPP-other). There was a significantly better clinical response rate from MPP-AS compared to MPP-other (87% vs. 65%, respectively, *p* = 0.003). The MPP-AS mode was more likely to convert non-responders to responders (100% vs. 49%, *p =* 0.014), possibly because the wide gap between the LV pacing poles increased the area of activated myocardium and probably by overcoming the likelihood of pacing across scar tissues [71]. A significant limitation of this study was that only patients demonstrating no haemodynamic compromise to MPP, as measured with echocardiography mitral flow VTI, were included and randomised. This potentially limited patient selection to long-term clinical “responders”; long-term data in a broad patient selection may be required. 

The MORE-CRT trial evaluated the effect of MPP on “non-responders”, defined as patients who demonstrated a <15% reduction in the LV end systolic volume (LVESV) compared to the baseline after 6 months of standard CRT [72]. These “non-responders” were randomly assigned to either continued conventional biventricular pacing or to MPP. The MPP cohort was further sub-randomised to the settings of a wide spatial separation with short (5 ms) intra- and interventricular timing delays (MPP-AS) and other (MPP-other). There was an equal proportion of conversion to “responders” within the two groups (MPP and biventricular pacing) after 6 months and no significant difference in the secondary endpoints (heart failure events, mortality and mean LVESV). A subgroup analysis of the MPP arm of the MORE-CRT trial revealed that the MPP-AS subgroup elicited a significantly higher conversion rate in comparison to the MPP-other group and produces a better proportion of “responders” than conventional biventricular pacing (45.6% vs. 33.8%, respectively). Based on these findings, the authors suggested that MPP itself requires optimisation with a wide anatomical separation and short timing intervals for maximal gain [72]. 

### 4.2. Adaptive Algorithms

An echocardiography-based optimisation to tailor the pacing settings to the individual patient yields clinical benefits, but it is time consuming and therefore costly [69]. An optimisation on a single occasion soon after implantation is unlikely to provide the ideal settings for the lifetime of the patient, because these are not static phenomena; the performance of the leads, the severity of the conduction system disruption and ventricular dysfunction can fluctuate or progress, and haemodynamic factors vary with autonomic changes in the short [55] and long term [73]. Proprietary device algorithms offer a form of optimisation that can be repeated frequently and automatically [74] (Table 1). Delnoy et al. demonstrated, in a cohort of 199 patients, that systematic regular optimisation using a proprietary algorithm (SonR^TM^, Microport, Shanghai, China) improved the event-free survival (*p* = 0.039) at a 1-year follow up compared to patients optimised less frequently [73]. 

#### 4.2.1. AdaptivCRT

The AdaptivCRT^®^ (Medtronic, Minneapolis, MN, USA) algorithm (aCRT) attempts to synchronise the intrinsic RV conduction with the activated LV, a concept based on the observation that LV pacing with normal AV conduction results in positive cardiac remodelling and improved biventricular function [75]. This is possible only in sinus rhythms with a normal intrinsic AV conduction (A_sense_ to RV_sense_ interval of ≤200 ms), which the algorithm assesses every minute. In other patients (a prolonged AV interval, heart rate > 100 bpm), adaptive biventricular pacing is activated, which the device attempts to optimise periodically through the AV/VV timings. 

Early evidence demonstrated that this algorithm was as effective as an echocardiography-guided optimisation: the adaptive CRT trial found a similar degree of improvement in Packer’s clinical composite score (NYHA class, patient global assessment, hospitalisation due to HF, all-cause mortality) and stroke volume (aortic velocity time integral) between this proprietary algorithm and an echocardiography-based optimisation [76]. More recent data show that synchronised LV pacing (sLVP) resulted in superior clinical outcomes; patients with a >50% sLVP had a reduced risk of HF hospitalisation and mortality than patients with a <50% sLVP [77]. 

The benefits of aCRT appear to result from synchronisation of the slow epicardial LV pacing with fast intrinsic RV activation, which diminishes RV pacing, a factor that has been shown to worsen dyssynchrony. The adaptive component of an aCRT algorithm also monitors and optimises the AV delay. The benefit of this algorithm compared to a traditional optimisation is that it facilitates resynchronisation during exercise; the AV delay plays a crucial role in synchronising LV pacing with the intrinsic RV activation during all physiological states [77], thereby maximising the duration of time patients maintain cardiac resynchronisation. Optimising the AV delay during exercise improves the LV filling time and exercise capacity. A trial of 52 patients demonstrated that the optimal AV delay had halved (49.5%) from rest to exercise in 94% of patients; optimisation of this index during exercise led to an improvement in the LV filling time, and when rate-adaptative AV delay was activated, patients exhibited an improved exercise capacity [78].

**Table 1:** Optimising methods in cardiac resynchronisation therapy.

Method	Description	Comments	Studies
**Non-echo guided**			
**Surface ECG**	VV interval: measurement of the narrowest QRS duration on surface ECG.	Simplest and widely available method. Can be combined with LVOT VTI measurements through echo.	Bertini et al. [53]: there was significant accordance with the echo-guided VV optimisation method.
**Intracardiac Electrograms**	AV/VV delay: estimated by the intrinsic interval delays during implantation.	Optimal VV delay= −0.333 × (RV − LV electrical delay) − 20 ms.	DECREASE-HF trial [59]: sequential BiV pacing with programmed LV and RV activation times in this way was similar to simultaneous BiV pacing.
**Invasive LV** ***dp/dtmax***	AV/VV delay: measurement of LV *dP/dt max* by a 0.014-in sensor-tipped pressure guidewire.	Invasive method. Not applicable for routine clinical follow up.	Van Gelder et al. [54]: significantly increased LV *dp/dt max* compared with simultaneous BiV pacing.
**Echo-guided**			
**LVOT VTI method**	AV/VV delay: optimal intervals correspond to the largest LVOT VTI.	Simple method. PW doppler used (in 20 ms steps) to determine optimal interval.	Bertini et al. [53]: Combined with surface ECG for a less-time optimisation approach.
**Iterative method**	AV delay: use of PW transmitral inflow to estimate maximal LV diastolic filling time.	No studies comparing this method to other optimising methods.	CARE-HF trial [18]: beneficial compared to medical treatments in terms of symptoms, quality of life and risk of death.
**Ritter’s method**	AV delay: use of two extreme delays (short and long) to determine the time between QRS onset and A-wave truncation.	Limited use in patients with a high HR or intrinsic AV interval < 150 ms. Validated only in patients with dual-chamber pacemakers and preserved LVEF.	Gold MR et al. [40]: inferior to electrogram-based optimisation. Melzer et al. [41]: good correlation with RNV in patients with an LVEF of <35%. Jansen AHM et al. [42]: no benefits observed in terms of invasive *dp/dt max* estimation.
**Device-related**			
**MPP-AS**	MPP optimisation: programming MPP with a wide LV electrode anatomical separation and short timing interval.	Long-term (6 months) clinical outcomes of this optimisation algorithm are debatable.	MPP IDE trial [71]: programming to pace from distal poles (MPP-AS) presents better clinical outcomes and is more likely to convert non-responders to responders. MORE-CRT MPP study [72]: MPP-AS subgroup experienced higher conversion rates to responders compared to MPP-other.
**AdaptivCRT** **^®^**	VV delay: synchronises the intrinsic RV conduction with activated LV. AV delay: monitors and optimises the AV delay.	Patients in sinus rhythm, with normal intrinsic AV conduction. Facilitates resynchronisation during exercise.	Adaptive CRT trial [76]: aCRT algorithm is safe and as effective as BiV pacing with comprehensive echo optimisation. Shanmugam et al. [78]: rate-adaptive AV delay during exercise improved exercise times and VO^2^_max_.
**CRT Autoadapt**	AV/VV delay: by comparing A-RV and A-LV intervals, BiV pacing configuration is determined.	Optimal AV delay: the shortest of “70% A-RV” or “A-RV–40 ms”. Not for patients with a complete AV block.	Trial (NCT04774523) is in progress, and the estimated completion is 2024.
**SyncAV** **^®^**	AV delay: monitors the intrinsic AV interval (every 256 beats) and optimises AV settings accordingly.	Based on the concept that AV delay is dynamic and should constantly be adjusted to stress and exercise.	Varma et al. [79]: in patients with LBBB, the SyncAV algorithm resulted in a significant reduction of the GRS duration regardless of PR, LV-paced intervals or underlying ischemic disease.
**SmartDelay (SD)**	AV delay: electrogram-based algorithm.	Adjust AV delay to changes in hemodynamic conditions.	SMART-AV trial [74]: SD optimisation was not different from echo-determined AV optimisation or a fixed AV delay of 120 ms.
**Peak Endocardial** **Acceleration Signals** **(PEAs)**	AV/VV delay: contractility-guided optimisation, using a sensor at the tip of the lead.	Optimisation based on the correlation of these endocardial signals with the cardiac cycle and the LV *dp/dt max*	RESPOND-CRT trial [80]: AV and VV optimisation was safe and as effective as echo-guided optimisation in increasing the response to CRT.

Abbreviations: ECG, electrocardiogram; VV, inter-Ventricular; LVOT VTI, left ventricular outflow tract velocity time integral; RV, right ventricle; LV, left ventricle; BiV, biventricular; AV, atrioventricular; PW, pulse wave; LVEF, left ventricular ejection fraction; HR, heart rate; RNV, radionuclide ventriculography; LBBB, left bundle branch block; MPP, multipoint pacing.

#### 4.2.2. SyncAV

SyncAV^®^ (Abbott medical, Abbott Park, IL, USA) is an algorithm that monitors the intrinsic AV interval (every 256 beats) and optimises AV settings accordingly. The AV delay is adjusted to 10–60 ms less than the measured interval. The ability to programme this offset interval from the intrinsic AV allows for patient-tailored programming, based on the understanding that intrinsic AV conduction, and therefore optimal AV delay, is dynamic and must constantly adapt to factors such as exercise and stress. 

A study investigating the effects of SyncAV^®^ with biventricular pacing found this combination to have a greater effect on the electrical resynchronisation (represented by a narrowing of the QRS complex) than nominal settings or by LV “fusion” pacing with intrinsic RV activation. Patient-tailored offset SyncAV^®^ with biventricular pacing recorded the narrowest QRS duration of all groups (123 ± 12 ms) and was the only proprietary setting to achieve 100% of patients with a narrower QRS than the baseline [79]. It was postulated that the abnormal depolarisation wavefront in patients requiring CRT cannot be rectified by the epicardial, non-apical pacing of LV-only pacing. Therefore, through a customised optimisation of the AV timings and biventricular pacing to produce an optimised paced wavefront, better synchronisation can be achieved, as indicated by the narrow QRS on the ECG [79]. 

Ventricular stimulation at the ideal sites and inter-electrode timing are reduced in value if the AV timing is suboptimal. In a study of 90 patients, the QRS duration was narrowed to a similar degree with a static AV delay with pacing from the latest-activated LV pole (138 ± 27 ms) and the earliest-activated LV pole (139 ± 26). By activating the SyncAV^®^ with a tailored offset, the QRS narrowed significantly further (123 ± 22 vs. 122 ± 24, respectively), highlighting the dominance of AV timing in achieving resynchronisation over inter- and intraventricular timings [81]. Further evaluation of this algorithm is taking place in the “Characterization of Acute and Long-Term Response to Left Ventricle Only Pacing Combined with MultiPoint Pacing and SyncAV” trial (clinicalTrials.gov Identifier NCT03567096). 

#### 4.2.3. SmartDelay

One of the first commercially available proprietary algorithms optimising CRT was SmartDelay (Boston Scientific, Marlborough, MA, USA). This algorithm was developed based on AV timing optimisation using intracardiac electrograms (EGM) and the effects of this on the LV *dp/dt max* [40]. This function utilises the intrinsic AV interval and the native interventricular timing to calculate the optimal AV delay (and the site of LV pacing) that will allow for the fusion between paced and intrinsic conduction. The added capability of modifying the settings according to atrial-sensed or atrial-paced beats offered an additional advantage over other forms of CRT optimisation. 

The SMART-AV trial randomised 980 patients to have AV delay optimisation with echocardiography (iterative method), the SmartDelay proprietary function or a fixed interval of 120 ms in a 1:1:1 fashion. This multi-arm randomised trial found that SmartDelay was non-inferior to echocardiography AV optimisation and that both interventions were similar in outcomes to the nominal 120 ms interval; there was no significant difference in quality of life, LVEF or changes in LV volumes between the three groups [74]. SmartDelay was advantageous over echocardiography for optimisation as it could be performed quickly, regularly and with limited resources to achieve the same outcomes. As the nominal fixed AV interval of 120 ms was non-inferior to the AV optimisation methods, it was suggested that CRT optimisation should be reserved for the non-responders; the nominal setting may be sufficient. 

SMART-CRT is a multi-centre randomised study, comparing SmartDelay and a fixed AV interval of 120 ms in patients requiring CRT with an interventricular delay of ≥70 ms. The authors found that a similar proportion of patients achieved >15% of LV reverse remodelling in the SmartDelay and the fixed AV delay cohorts; the lack of difference was credited to the improvement in HF medical therapy. The significant finding was that, in patients with a prolonged interventricular delay, SmartDelay contributes to a significantly higher degree of reverse modelling than the fixed AV interval [82]. From a device perspective, finetuning the sensed and paced AV delays is crucial to optimising the AV interval; the atrial-sensed AV delay is shorter than the atrial-paced AV delay. This effects the timing of the LA contraction and subsequent LV filling. Kloosterman et al. demonstrated a simple and effective IEGM-based method of optimising the AV delay by calculating the difference between the paced and sensed AV delays. In 328 patients, the authors demonstrated that the optimal AV delay calculated using this simple method correlated with the echocardiography optimisation AV delay and that the CRT responders had almost zero differences between these two methods; the paced AV delay optimisation is an important component to resynchronisation [83]. 

#### 4.2.4. CRT AutoAdapt

The CRT AutoAdapt (BIOTRONIK, Berlin, Germany) algorithm encourages LV-only pacing to provide an optimal interventricular fusion between the intrinsic RV depolarisation with the paced wavefront. This function evaluates the AV interval by measuring the A-RV and A-LV timings; based on this evaluation, the device is able to configure LV-only pacing for a fusion with the intrinsic conduction or biventricular pacing. The algorithm is designed to evaluate the AV interval every minute and configure the ventricular pacing accordingly. This is based on the concept that the AV interval is dynamic; frequent assessments of the intrinsic AV timings should permit the device to adapt to the varying AV delays and ensure that resynchronisation is optimal. The requirements of this function are sinus rhythms, an atrial rate of <100 beats per minute and an A-RV conduction after pace of <250 ms. The BIO|Adapt study is currently recruiting to assess the outcomes of this algorithm and is due to complete in 2024 (clinicalTrials.gov Identifier NCT04774523). 

#### 4.2.5. Peak Endocardial Acceleration Signals

Ventricular contraction generates mechanical vibrations which propagate through the myocardium. These vibrations can be measured using an endocardial accelerometer at the tip of pacing leads, which processes them as Peak Endocardial Acceleration (PEA) signals. These signals correlate with LV *dp/dt max*, usable as an index of cardiac contractility [84]. PEA amplitudes and the heart sounds relate with one another [85], with the highest amplitude generated by the isovolumetric contraction–closure of the AV valves as the ventricular pressure accelerates [86]. A pacing lead equipped to measure these signals can recognise the phases of the cardiac cycle and optimise the AV/VV timing intervals. 

The RESPOND-CRT trial used the SonRtip™ (Microport, China) atrial lead in CRT to optimise the AV/VV settings on a weekly basis and compared the outcomes with an echocardiography-based optimisation. This non-inferiority trial demonstrated a statistically similar proportion of responders in the SonRtip™ and echocardiogram-optimisation groups (75% vs. 70.4%, *p* < 0.001), concluding that this proprietary algorithm is non-inferior to echocardiography optimisation. There were also similar rates of HF-related hospitalisations and deaths between the two groups (14.2% vs. 17.6%, respectively, *p* < 0.001) [80]. 

## 5. Conduction System Pacing 

His bundle pacing (HBP) and Left bundle branch pacing (LBBP) are important recent innovations in cardiac resynchronization therapy. In principle, using the conduction system should be better than any form of biventricular pacing. By recruiting the intrinsic conduction system, it provides a complete restoration of the physiological biventricular activation, overcoming all forms of atrioventricular and intraventricular delays. Recent experience has shown that even in patients with a marked intraventricular conduction delay, the distal conduction system is often well preserved and can be utilised by stimulation at a site downstream of damaged areas in the proximal conduction system. Even if the downstream conduction system later fails, the septal pacing site used in conduction system pacing is ideally situated between the right ventricle and the latest-activated segment of the lateral LV; activation from this “midpoint” should provide a homogenous dispersion of the depolarisation wavefront (Figure 2). To this effect, the value of conduction system stimulation was visualised in a small study of 10 patients by means of electrocardiographic imaging (Medtronic, Minneapolis, MN, USA); simulated LBBP using a decapolar catheter resulted in a homogenous epicardial activation of the LV, whilst biventricular pacing produced the earliest activation at the LV lateral wall [49].

The feasibility of the conduction system was first determined by Deshmukh et al. In 14 patients with a dilated cardiomyopathy (DCM) and normal QRS, they were able to permanently pace the His bundle with resulting improvements in cardiac function and functional status [87] over a 2-year follow-up period (23.4 ± 8.3 months). Huang et al. subsequently demonstrated that patients with an impaired LV function (DCM) and LBBB gained significant benefits from HBP. In 56 patients who had received permanent HBP, the paced QRS was significantly narrowed, and after a 3-year follow-up period, there were notable improvements in the LV function and NYHA class. When compared to 15 patients who received biventricular pacing, the authors found no significant differences in the clinical outcomes, although this study was small with a low power. The authors concluded that conduction-based pacing could be an alternative option to biventricular pacing for CRT [88]. 

There are important limitations often associated with HBP including a poor pacing stability, a low success rate of implantation or the presence of conduction blocks close to the His level. To overcome these limitations, LBBP can be deployed to pace distal to the His within the interventricular septum. Huang et al. successfully performed LBBP in 63 patients with LBBB and DCM, achieving a 97% implant rate. There was an immediate correction of the QRS duration, and over the 18-month follow-up period, there was a significant improvement in the LV function, which was reflected in the improvement in the NYHA class. Importantly, the pacing indices remained stable over this period. This small study demonstrated the feasibility, stability and efficacy of LBBP in CRT [89]. 

In a small, randomised trial of 40 heart failure patients (non-ischaemic cardiomyopathy) with CRT indications, Wang et al. compared biventricular pacing with LBBP. In the by per-protocol analysis, the QRS duration was narrower with LBBP (138.5 ± 10.6 vs. 129.2 ± 10.8 ms, respectively) although not it did not reach statistical significance, but after a 6-month follow-up period, LBBP resulted in a significantly greater improvement in the LVEF [90]. Although this small pilot study lacked the power to provide firm conclusions, it indicated a favourable improvement in the LVEF associated with LBBP. A perceived limitation of biventricular pacing in these early comparative studies is the lack of device optimisation. Chen et al., in a prospective observational study of 100 HF patients with LBBB, compared LBBP with optimised biventricular pacing using the aCRT proprietary algorithm. In comparison to aCRT, LBBP had resulted in a narrower QRS duration and a significantly greater improvement in the LVEF (6 months and 12 months). Clinically, whilst the proportion of patients in the NYHA class III–IV decreased in both groups of CRT, LBBP resulted in a higher proportion of patients with an improvement in the NHYA class than aCRT over the 6-month and 1-year follow up [91]. Due to the small population size and observational data, these studies can be considered as hypothesis generating, increasing the need for larger, randomised trials to compare CRT modalities. 

## 6. Conclusions

The optimisation of the pacing site, the optimisation of the pacing modality and the optimisation of device settings are significant components of resynchronisation therapy. The echocardiography-based method of device settings is regarded as the “gold standard”, but it is resource intensive and user dependent. The large number of programmable variables available on the more sophisticated CRT devices makes it impossible to test all options echocardiographically to select the best. Proprietary algorithms now provide an alternative or supplementary method of identifying the best settings, and evidence suggests that they are at least equal to the traditional method. Their potential is much greater than this, as algorithms are not limited to a single or occasional optimisation but can be repeated frequently to adapt to changing conditions. With a variety of programs now available, the picture is unclear; the answer may lie in selecting the most appropriate algorithm for each individual, optimising the optimisation to suit the patient.

## Data Availability

Not applicable.
